# Beta-Adrenergic Receptor Stimulation Modulates the Cellular Proarrhythmic Effects of Chloroquine and Azithromycin

**DOI:** 10.3389/fphys.2020.587709

**Published:** 2020-10-22

**Authors:** Henry Sutanto, Jordi Heijman

**Affiliations:** Department of Cardiology, Cardiovascular Research Institute Maastricht (CARIM) School for Cardiovascular Diseases, Maastricht University, Maastricht, Netherlands

**Keywords:** arrhythmia, computational modeling, COVID-19, chloroquine, azithromycin, beta-adrenergic, electrophysiology-basic

## Abstract

The antimalarial drug, chloroquine (CQ), and antimicrobial drug, azithromycin (AZM), have received significant attention during the COVID-19 pandemic. Both drugs can alter cardiac electrophysiology and have been associated with drug-induced arrhythmias. Meanwhile, sympathetic activation is commonly observed during systemic inflammation and oxidative stress (e.g., in SARS-CoV-2 infection) and may influence the electrophysiological effects of CQ and AZM. Here, we investigated the effect of beta-adrenergic stimulation on proarrhythmic properties of CQ and AZM using detailed *in silico* models of ventricular electrophysiology. Concentration-dependent alterations in ion-channel function were incorporated into the Heijman canine and O’Hara-Rudy human ventricular cardiomyocyte models. Single and combined drug effects on action-potential (AP) properties were analyzed using a population of 1,000 models accommodating inter-individual variability. Sympathetic stimulation was simulated by increasing pacing rate and experimentally validated isoproterenol (ISO)-induced changes in ion-channel function. In the canine ventricular model at 1 Hz pacing, therapeutic doses of CQ and AZM (5 and 20 μM, respectively) individually prolonged AP duration (APD) by 33 and 13%. Their combination produced synergistic APD prolongation (+161%) with incidence of proarrhythmic early afterdepolarizations in 53.5% of models. Increasing the pacing frequency to 2 Hz shortened APD and together with 1 μM ISO counteracted the drug-induced APD prolongation. No afterdepolarizations occurred following increased rate and simulated application of ISO. Similarly, CQ and AZM individually prolonged APD by 43 and 29% in the human ventricular cardiomyocyte model, while their combination prolonged APD by 76% without causing early afterdepolarizations. Consistently, 1 μM ISO at 2 Hz pacing counteracted the drug-induced APD prolongation. Increasing the I_Ca,L_ window current produced afterdepolarizations, which were exacerbated by ISO. In both models, reduced extracellular K^+^ reduced the repolarization reserve and increased drug effects. In conclusion, CQ- and AZM-induced proarrhythmia is promoted by conditions with reduced repolarization reserve. Sympathetic stimulation limits drug-induced APD prolongation, suggesting the potential importance of heart rate and autonomic status monitoring in particular conditions (e.g., COVID-19).

## Introduction

Nine-months after its first identification in Wuhan, China in December 2019, severe acute respiratory syndrome-associated coronavirus type-2 (SARS-CoV-2) infection [i.e., coronavirus disease 2019 (COVID-19)] has contributed to more than 850,000 deaths worldwide and has been declared a pandemic with significant global socioeconomic impact (Nicola et al., 2020). At the moment, the exact pathophysiology of the disease remains unclear and no definitive therapy is available. Several drugs are considered effective in preclinical studies and are currently being tested against SARS-CoV-2 in the clinic (e.g., the antivirals lopinavir, ritonavir, and remdesivir; the antimicrobial azithromycin; the antimalarial drugs, chloroquine and hydroxychloroquine, and more recently antiparasitic ivermectin; Caly et al., 2020; Lu et al., 2020; Wang et al., 2020b). Of those, chloroquine (CQ) and azithromycin (AZM) have gained significant attention due to their high accessibility and low cost. Nonetheless, their effectivity against COVID-19 has not been confirmed by any large clinical trial and their use is controversial. Some studies reported the benefit of those drugs (Arshad et al., 2020; Colson et al., 2020; Gao et al., 2020; Huang et al., 2020), while others reported no effect (Borba et al., 2020; Mehra et al., 2020; Molina et al., 2020). This controversy is further complicated by the retraction of papers demonstrating the absence of benefit of these drugs in COVID-19 (Mehra et al., 2020), potential issues with study design and analysis (Atkinson, 2020; Varisco et al., 2020), and the termination of their emergency use by the United States Food and Drug Administration (FDA) due to their potential proarrhythmic effects (Uzelac et al., 2020).

Chloroquine is a widely-used antimalarial drug that inhibits multiple cardiac ion-channels (Crumb et al., 2016). It has been suggested to prevent the viral entry, transport, and post-entry events in COVID-19, most likely *via* its effects on endosomal pH and the resulting under-glycosylation of angiotensin-converting enzyme 2 (ACE2) receptors that are required for viral entry, although the exact mechanisms remain incompletely understood (Roden et al., 2020; Singh et al., 2020). Meanwhile, AZM is a broad-spectrum macrolide antibiotic that is believed to potentiate the effect of CQ, reducing the replication capabilities of SARS-CoV-2 (Singh et al., 2020). Similar to CQ, AZM also inhibits multiple cardiac ion-channels in a dose-dependent manner (Crumb et al., 2016). Therefore, the administration of CQ and AZM, alone or in combination, can prolong the ventricular cardiomyocyte action potential (AP) duration (APD) and, thereby, the QT interval on the electrocardiogram. Experimentally, the APD-prolonging effect of CQ and AZM, alone and in combination, has been shown in an anesthetized guinea-pig model (Fossa et al., 2007). Moreover, excessive QT-interval prolongation has been implicated in drug-induced malignant arrhythmias, such as torsade de pointes, by promoting early afterdepolarizations (EADs) and a heterogeneous repolarization substrate, and consistently observed in COVID-19 patients with CQ and AZM (Chorin et al., 2020; Jankelson et al., 2020; Saleh et al., 2020; Uzelac et al., 2020; van den Broek et al., 2020).

**Figure 1:**
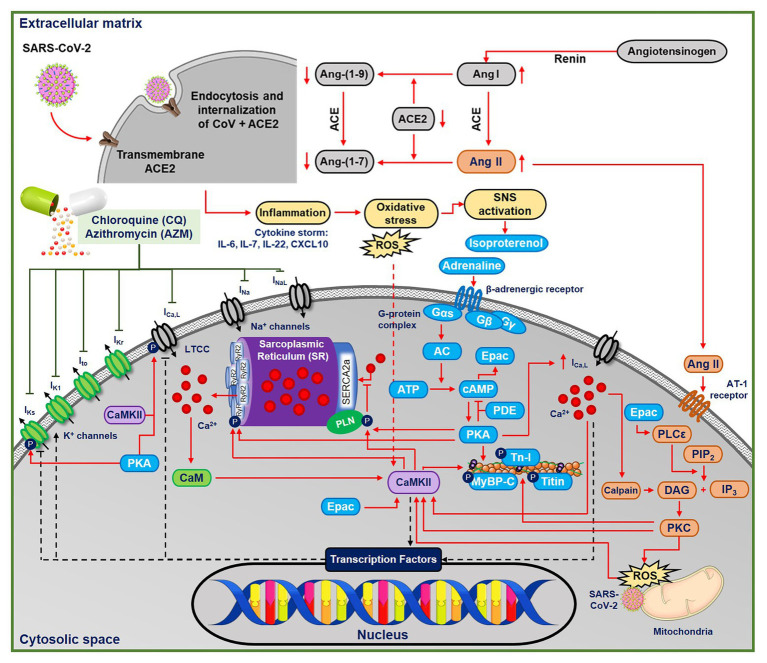
The multifactorial effects of coronavirus disease 2019 (COVID-19) in the ventricular cardiomyocyte and the ionic targets of chloroquine (CQ) and azithromycin (AMZ). The severe acute respiratory syndrome-associated coronavirus type-2 (SARS-CoV-2) leads to the endocytosis and internalizations of the transmembrane angiotensin converting enzyme type 2 (ACE2) receptors, preventing the conversion of angiotensin I and II into their metabolites. Thus, angiotensin II binds to the AT-II receptor, initiating protein kinase-C (PKC)-dependent pathways, which may further activate Ca^2+^/calmodulin-dependent protein kinase II (CaMKII)-dependent signaling cascades. In COVID-19, the systemic inflammation and cytokine storm can also increase oxidative stress, leading to reactive oxygen species (ROS)-mediated CaMKII activation. CQ and AZM alter action potential properties through inhibition of multiple cardiac ion channels [fast Na^+^ current (I_Na_), late Na^+^ current (I_NaL_), rapid delayed-rectifier K^+^ current (I_Kr_), slow delayed-rectifier K^+^ current (I_Ks_), inward-rectifier K^+^ current (I_K1_), transient-outward K^+^ current (I_to_), and L-type Ca^2+^ current (I_Ca,L_)]. AC, adenylyl cyclase; ACE, angiotensin converting enzyme; Ang II, angiotensin II; ATP, adenosine triphosphate; CaM, calmodulin; CaMKII, Ca^2+^/calmodulin-dependent protein kinase II; cAMP, cyclic adenosine monophosphate; DAG, diacyl glycerol; IL, interleukin; IP_3_, inositol triphosphate; PDE, phosphodiesterase; PIP_2_, phosphatidylinositol biphosphate; PKA, protein kinase A; PKC, protein kinase C; PLC, phospholipase C; ROS, reactive oxygen species; Tn-I, troponin-I.

Although the cardiac pathophysiology of COVID-19 remains incompletely understood, several aspects point toward increased incidence of cardiac arrhythmias (Bhatla et al., 2020). SARS-CoV-2 induces systemic inflammation, leading to cytokine storm (Lazzerini et al., 2020), which is expected to increase oxidative stress by releasing reactive oxygen species (ROS). Moreover, CQ may itself promote oxidative stress (Murphy et al., 2019). Both inflammation and oxidative stress have been associated with increased arrhythmogenic risk (Adameova et al., 2020), e.g., through activation of Ca^2+^/calmodulin-dependent protein kinase II (CaMKII; Swaminathan et al., 2012; Wang et al., 2018) and NLRP3 inflammasome (Monnerat et al., 2016), as observed in COVID-19 (Shah, 2020). Troponin-T, a marker for myocardial injury, has been shown to increase in groups of COVID-19 patients with malignant arrhythmias, denoting the major detrimental impact of SARS-CoV-2 on the cardiovascular system (Nishiga et al., 2020). Moreover, COVID-19 may activate the beta-adrenergic signaling cascade in cardiomyocytes *via* the stimulation of the sympathetic nervous system (Lazzerini et al., 2020). Palpitation, which is commonly associated with the activation of beta-adrenergic response, has been reported to be the main symptom of COVID-19 (Nishiga et al., 2020). Altogether, these processes may increase the propensity for cardiac arrhythmias by altering cardiomyocyte Ca^2+^-handling and modulating ion-channel properties (Figure 1; Sutanto et al., 2020b).

To the best of our knowledge, previous experimental and observational studies of the potential proarrhythmic effects of CQ and AZM have not considered the role of beta-adrenergic receptor stimulation. Computational modeling has increasingly been used in cardiac safety pharmacology to predict the proarrhythmic effect of novel compounds (Qu et al., 2019; Li et al., 2020; Ridder et al., 2020). Therefore, this study aimed to assess the potential cellular proarrhythmic effects of CQ and AZM in both the absence and presence of beta-adrenergic receptor stimulation using a population of detailed *in silico* models of ventricular electrophysiology.

## Materials and Methods

**Figure 2:**
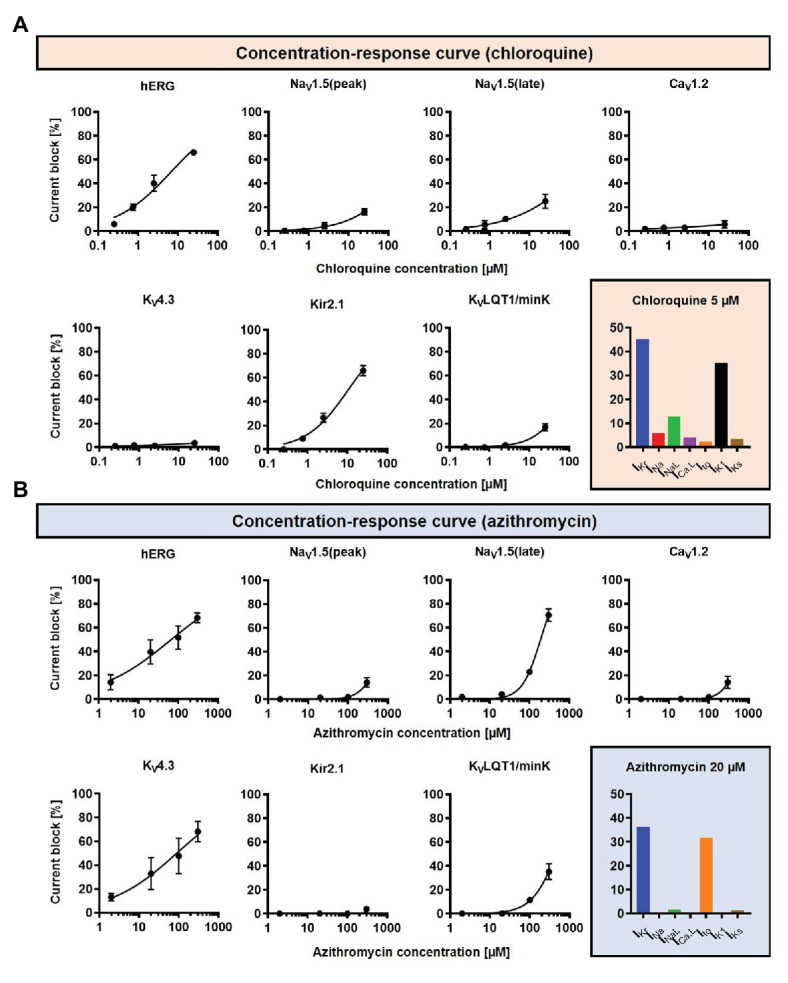
The concentration-dependent effects of CQ and AZM on cardiac ion channels. **(A)** CQ mainly blocks I_Kr_ and I_K1_, with minor effects on I_Na_, I_NaL_, I_to_, I_Ca,L_, and I_Ks_. **(B)** AZM mainly blocks I_Kr_ and I_to_, with minimal effects on I_Na_, I_NaL_, I_Ca,L_, I_K1_, and I_Ks_. The experimental data (black symbols) were obtained from previous experiments (Crumb et al., 2016) and were fitted using Hill equations in the model (black lines). Bar charts show percentage inhibition of different ion channels using the clinically relevant concentrations employed in subsequent simulations.

Concentration-dependent CQ and AZM-induced alterations in seven ion-channels [fast Na^+^ current (I_Na_), late Na^+^ current (I_NaL_), L-type Ca^2+^ current (I_Ca,L_), transient-outward K^+^ current (I_to_), inward-rectifier K^+^ current (I_K1_), rapid delayed-rectifier K^+^ current (I_Kr_), and slow delayed-rectifier K^+^ current (I_Ks_); Crumb et al., 2016; Figure 2] were incorporated into the Heijman canine ventricular cardiomyocyte model (Heijman et al., 2011) with beta-adrenergic receptor signaling and O’Hara-Rudy (ORd) human ventricular epicardial cardiomyocyte model (O’Hara et al., 2011). The Heijman canine ventricular model was employed in this study because it is one of the few cardiomyocyte models of a large mammal incorporating detailed beta-adrenergic signaling cascades, including PKA- and CaMKII-mediated phosphorylation of cardiac ion channels. Meanwhile, the ORd human ventricular model was chosen because it is the most widely used human ventricular cardiomyocyte model that works over a wide range of experimental conditions. To simulate the electrophysiological effects of beta-adrenergic stimulation in the human ventricular model, the experimentally validated changes in ionic current properties in response to 1 μM ISO (O’Hara and Rudy, 2012a) were applied to the ORd model and further calibrated based on recent data (Gong et al., 2020) to improve the model accuracy during maximal ISO stimulation (1 μM).

A drug concentration within the therapeutic range of CQ and AZM was selected (5 and 20 μM, respectively; Gielen et al., 2010; Wang et al., 2020a) and cellular simulations were performed in Myokit (Clerx et al., 2016). The effects of the drugs, alone and in combination (assuming independent drug-binding sites), on AP properties were assessed. Sympathetic stimulation was simulated by an increase in pacing rate and experimentally validated isoproterenol (ISO)-induced changes in ion-channel function (Heijman et al., 2011; Tomek et al., 2017). All results are presented during steady-state pacing at the indicated pacing frequencies (after 1,000 beats of prepacing). To evaluate the robustness of our findings and assess potential consequences of intra- and inter-subject variability on the electrophysiological effect of CQ and AZM, the maximum conductance of nine major ionic currents (I_Na_, I_NaL_, I_Ca,L_, I_Kr_, I_Ks_, I_K1_, I_to_, I_NCX_, and I_NaK_) were scaled based on a normal distribution with mean 1.0 and standard deviation 0.2, to create populations of models, as previously described (Sobie, 2009; Sutanto et al., 2020a). In brief, 1,000 variants of the model were created and the variants displaying “non-physiological” AP properties were excluded. Non-physiological was defined as APD_90_ or RMP outside the range of 3 standard deviations of experimental APD_90_ and RMP from Anyukhovsky et al. (1996) and Britton et al. (2017), based on previous studies (Sanchez et al., 2014; Britton et al., 2017). In total, 592 out of 1,000 canine ventricular models and all 1,000 human ventricular models were included. The non-normally distributed data are presented as median and inter-quartile ranges (IQR). The model code is available at www.github.com/jordiheijman.

## Results

### The Effects of Chloroquine and Azithromycin on Canine Ventricular Electrophysiology

**Figure 3:**
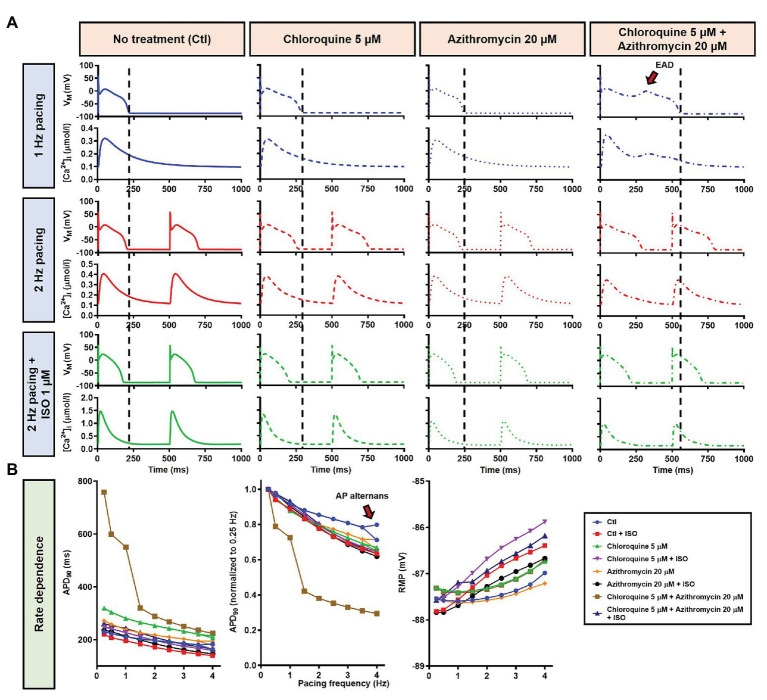
The effects of CQ and AZM on action potential (AP) properties of canine ventricular epicardium. **(A)** The AP and Ca^2+^ transient of non-treated, CQ 5 μM, AZM 20 μM, and combined groups. The dashed vertical lines indicate the end of the AP with 1 Hz pacing to provide a clearer depiction of the effects of increasing pacing rate and isoproterenol (ISO) on action potential duration (APD). The early afterdepolarization (EAD) is indicated with an arrow. **(B)** APD and resting membrane potential (RMP) for different pacing rates in the four groups with and without simulated beta-adrenergic stimulation. The occurrence of alternans at 4 Hz pacing is marked with an arrow. AP, action potential; APD, action potential duration; Ctl, control; ISO, isoproterenol; RMP, resting membrane potential.

**Figure 4:**
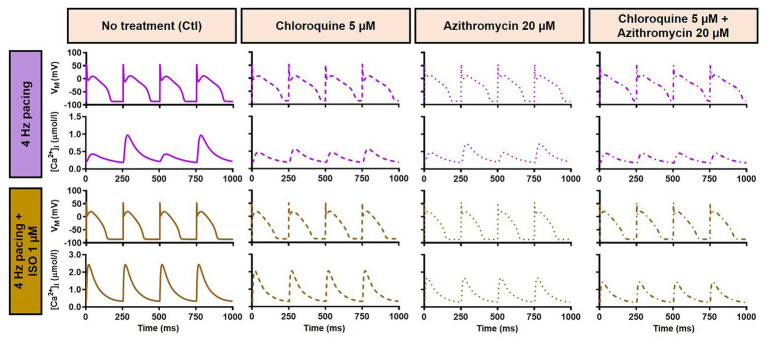
Action potential and Ca^2+^-transient alternans at 4 Hz pacing. The **upper panels** exemplify the AP and Ca^2+^-transient alternans at 4 Hz pacing in non-treated, CQ alone, and AZM alone groups. The application of 1 μM ISO abolishes the alternans, as shown in the **lower panels**. AP, action potential; AZM, azithromycin; Ctl, control; CQ, chloroquine; ISO, isoproterenol.

During 1 Hz pacing, application of 5 μM CQ in the Heijman canine ventricular epicardial cardiomyocyte model prolonged APD by 70 ms (+33%), while 20 μM AZM prolonged APD by 27 ms (+13%). The combination of both drugs showed a synergistic effect with an APD prolongation of 339 ms (+161%) and the occurrence of an EAD, as shown in Figure 3A, upper panels. Subsequently, the contributions of beta-adrenergic-dependent signaling cascades were assessed in two ways: by increasing the pacing frequency and through the simulated application of a maximal concentration of the beta-adrenergic receptor agonist ISO (1 μM) in combination with the escalation of pacing rate. Increasing the pacing rate from 1 to 2 Hz reduced the APD in all groups, with APD reduction of 14 ms (−7%) in the non-treated, 28 ms (−10%) in the CQ, 21 ms (−9%) in the AZM, and 261 ms (−47%) in the combined groups. The previously observed EAD in the combined group was not observed following the increase in pacing rate (Figure 3A, middle panels). The combination of simulated ISO application and increased pacing rate further reduced APD, with APD reduction of 40 ms (−19%) in the non-treated, 84 ms (−30%) in the CQ, 53 ms (−22%) in the AZM, and 341 ms (−62%) in the combined groups compared to APD during 1 Hz pacing (Figure 3A, lower panels). Increasing the pacing rate up to 4 Hz further reduced APD and lowering the pacing rate from 1 to 0.25 Hz prolonged the APD and resulted in EADs in the combined CQ + AZM group (Figure 3B, left and middle panels). At 4 Hz, Ca^2+^-transient and AP alternans was observed in the non-treated, CQ alone, and AZM alone groups, and its occurrence was prevented in the presence of ISO (Figure 4). Furthermore, at pacing rates >1 Hz, AZM slightly hyperpolarized the RMP, which was opposed by the RMP-depolarizing effect of beta-adrenergic activation, while CQ with or without ISO consistently showed a slight depolarization of RMP, likely due to its inhibition of I_K1_ (Figure 2). The RMP modulating effect was attenuated at slow pacing rates (Figure 3B, right panel).

**Figure 5:**
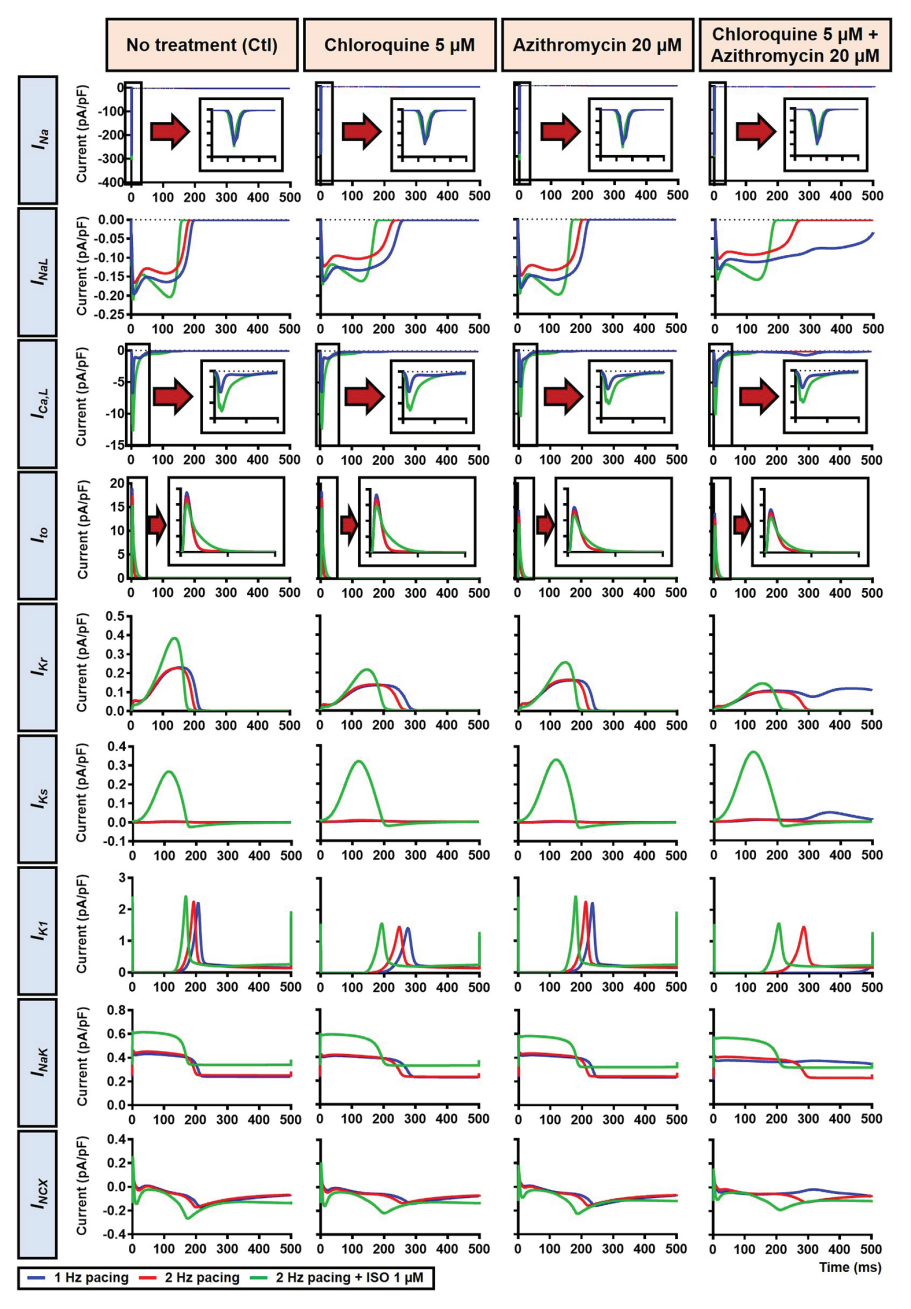
The effects of beta-adrenergic stimulation on cardiac ion channels of canine ventricular epicardium. The effects were assessed in four groups: non-treated, CQ, AZM, and combined groups. The blue lines represent the ionic currents during 1 Hz pacing, the red lines represent the ionic currents during 2 Hz pacing, and the green lines represent the currents during 2 Hz pacing with ISO 1 μM. The fast Na^+^ current (I_Na_), L-type Ca^2+^ current (I_Ca,L_), and transient-outward K^+^ current (I_to_) are shown at an expanded scale in the insets. Ctl, control; ISO, isoproterenol.

**Figure 6:**
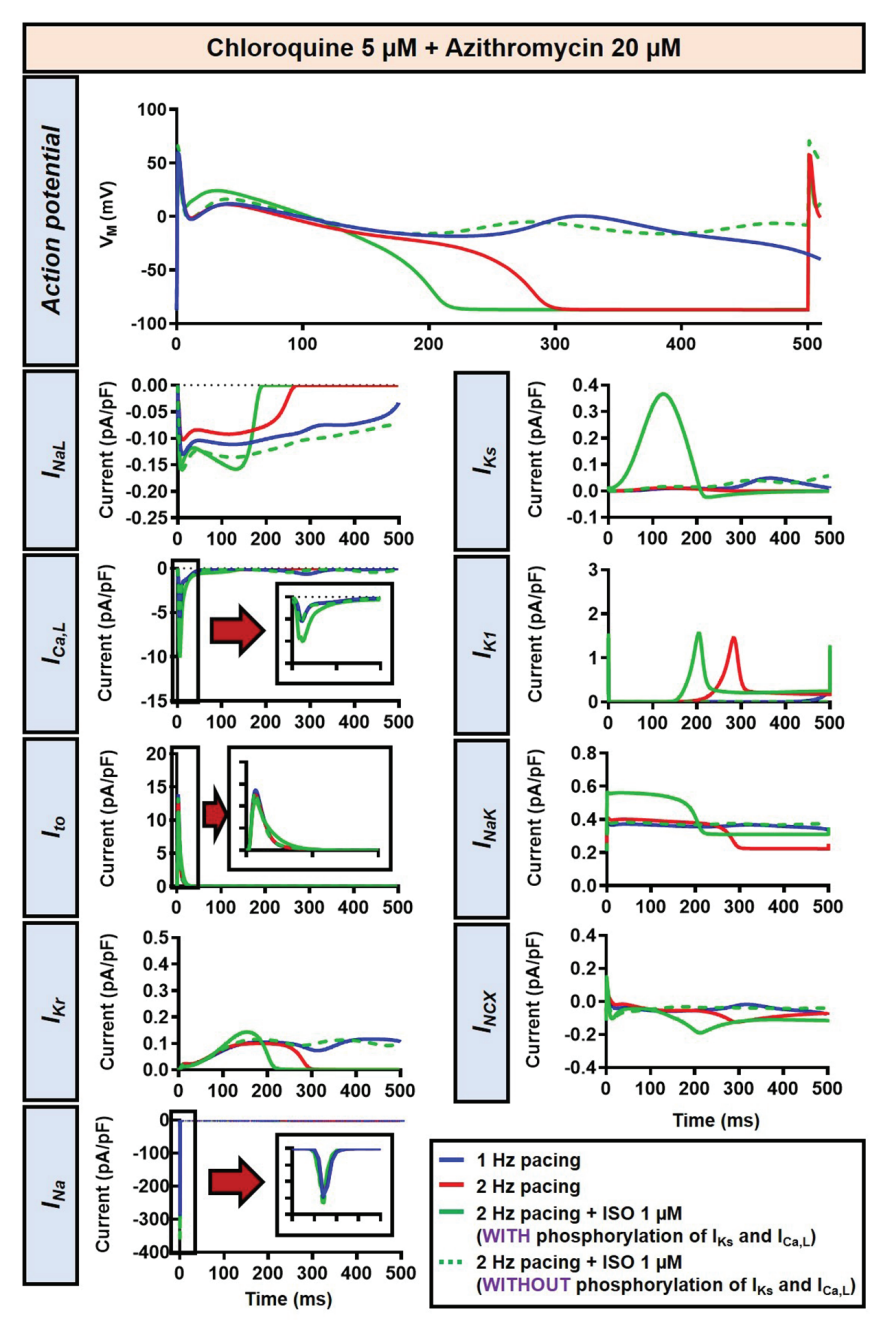
The impact of I_Ks_ and I_Ca,L_ phosphorylation on the action potential of canine ventricular epicardium in the presence of CQ and AZM. The effect of CQ 5 μM in combination with AZM 20 μM in the presence of ISO-induced I_Ks_ and I_Ca,L_ phosphorylation is shown in solid green lines. The green dashed lines represent the effect of CQ 5 μM in combination with AZM 20 μM in the absence of ISO-induced I_Ks_ and I_Ca,L_ phosphorylation.

The beta-adrenergic-induced modification of nine ionic currents (I_Na_, I_NaL_, I_Ca,L_, I_to_, I_Kr_, I_Ks_, I_K1_, I_NCX_ and I_NaK_) can be seen in Figure 5, highlighting the significantly increased I_Ks_ during beta-adrenergic stimulation. Indeed, ISO-induced phosphorylation of I_Ks_ and I_Ca,L_ was key for the observed APD reduction in the model, as previously documented (Heijman et al., 2011) and preventing such phosphorylation resulted in repolarization failure (RF) in the CQ + AZM group in the presence of simulated beta-adrenergic stimulation (Figure 6).

**Figure 7:**
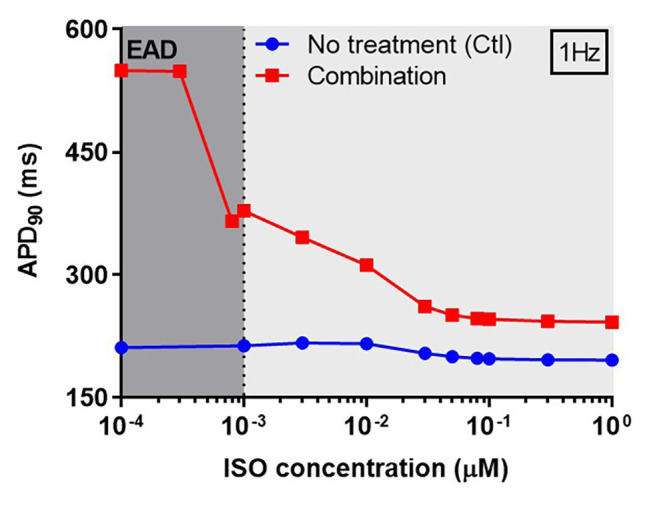
The concentration-dependent effect of ISO on APD of canine ventricular epicardium. The APD in the presence of various concentrations of ISO from 0.1 nM to 1 μM in non-treated group (blue line) was compared to the combined group (CQ 5 μM + AZM 20 μM; red line). The simulations were performed with 1 Hz pacing. APD, action potential duration; Ctl, control; ISO, isoproterenol.

**Figure 8:**
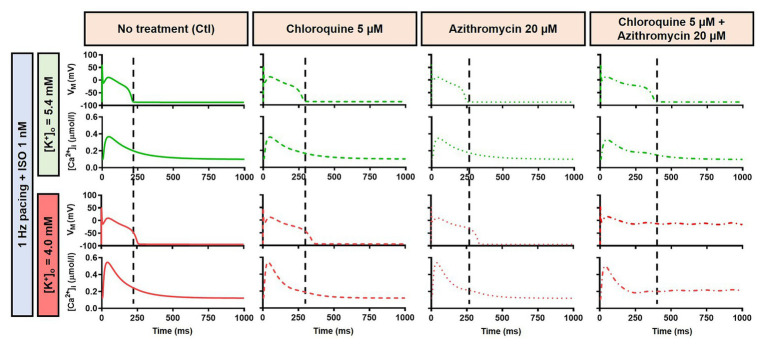
The effects of CQ and AZM on canine AP in the presence of an intermediate concentration of ISO. The AP and Ca^2+^ transient of non-treated, CQ 5 μM, AZM 20 μM and combined groups with 1 Hz pacing and 1 nM ISO in the presence of low extracellular K^+^ ([K^+^]_o_ = 4.0 mM) were compared to [K^+^]_o_ = 5.4 mM. The dashed vertical lines indicate the end of AP in models with [K^+^]_o_ = 5.4 mM to provide a clearer depiction of the effects of reduced extracellular K^+^ on APD. AP, action potential; APD, action potential duration; Ctl, control; ISO, isoproterenol.

Next, we investigated concentration-dependent effects of ISO on APD in both non-treated (control) and CQ + AZM groups at 1 Hz pacing. In the control group, ISO produced a mild APD shortening at concentrations >10 nM, as previously demonstrated (Heijman et al., 2011). In the combined CQ + AZM group, low concentrations of ISO up to 0.3 nM did not prevent EADs. At 0.3–1.0 nM, ISO abbreviated APD and diminished the occurrence of EADs. Further increasing the ISO concentration to 50 nM produced a progressive reduction in APD from 378 ms at 1 nM to 250 ms at 50 nM (−34%). ISO concentrations >50 nM produced minor additional changes in APD (Figure 7). The cellular effect of 1 nM ISO in all four treatment groups can be seen in Figure 8, upper panels.

**Figure 9:**
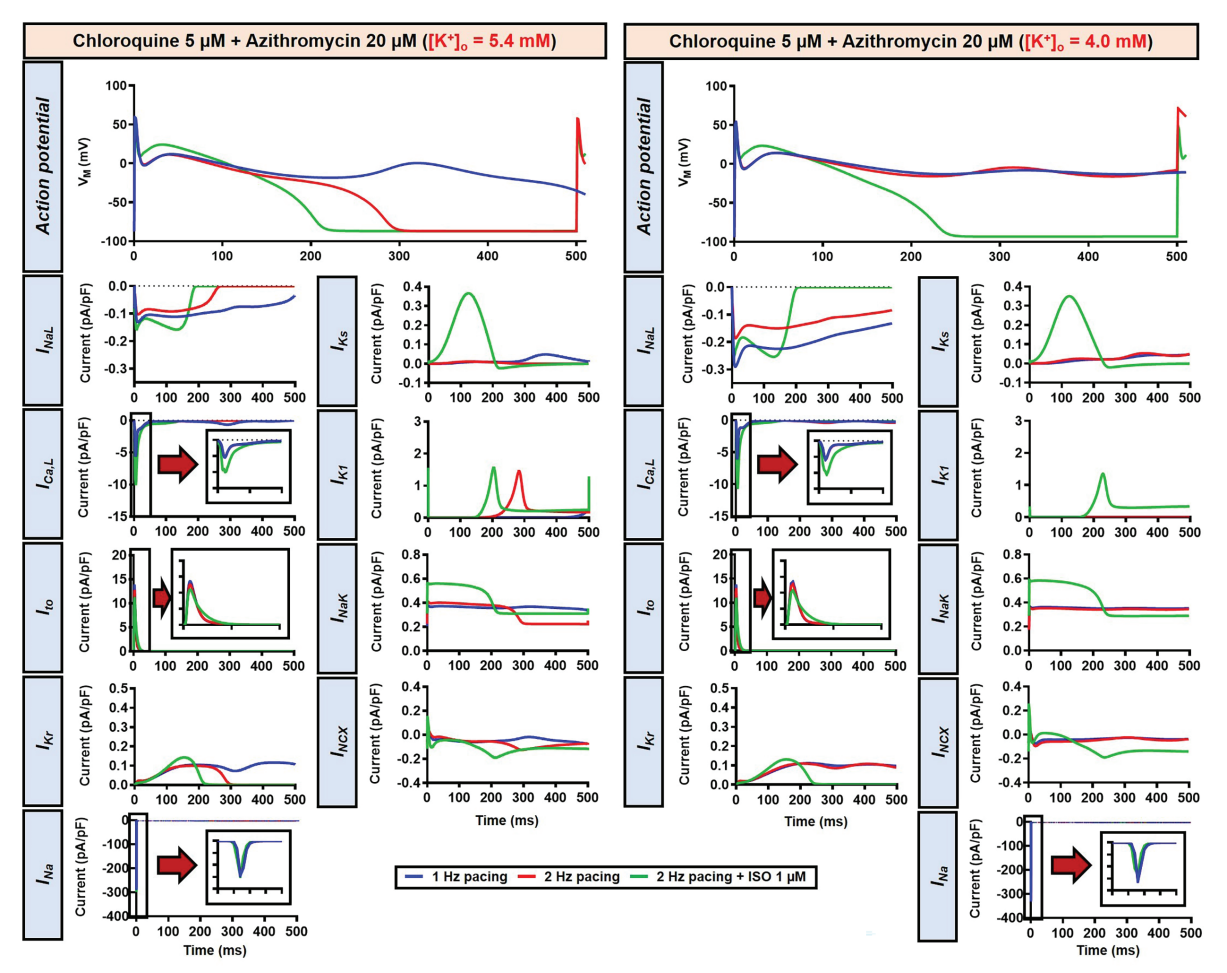
The impact of reduced extracellular K^+^ on the action potential of canine ventricular epicardium in the presence of CQ and AZM. The **left panels** show the effect of CQ 5 μM in combination with AZM 20 μM with 5.4 mM [K^+^]_o_. The **right panels** show the effect of CQ 5 μM in combination with AZM 20 μM in the presence of reduced [K^+^]_o_ (4.0 mM).

Alteration of cardiac repolarization by a pharmacological agent is frequently observed. However, sole administration of a drug rarely induces arrhythmias unless other predisposing factors aggravate the proarrhythmic risk by challenging the repolarization reserve (e.g., preexisting disease, concomitant drugs, or hypokalemia). We employed the Heijman canine ventricular cardiomyocyte model to investigate the effect of alterations to repolarization reserve, particularly in the setting of low extracellular K^+^ (hypokalemia). As shown in Figure 9, the simulated application of CQ + AZM with [K^+^]_o_ of 4.0 mM (instead of the default 5.4 mM) prolonged APD and caused RF in the absence of ISO and slight APD prolongation with 1 μM ISO. Moreover, the RMP was slightly hyperpolarized in the presence of hypokalemia. With 4.0 mM extracellular K^+^, higher concentrations of ISO are needed to restore these repolarization abnormalities: 1 nM ISO prevents EADs and RF with 5.4 mM extracellular K^+^ but not with 4.0 mM (Figure 8). These data highlight the importance of baseline repolarization reserve for the cellular proarrhythmic effects of CQ and AZM.

**Figure 10:**
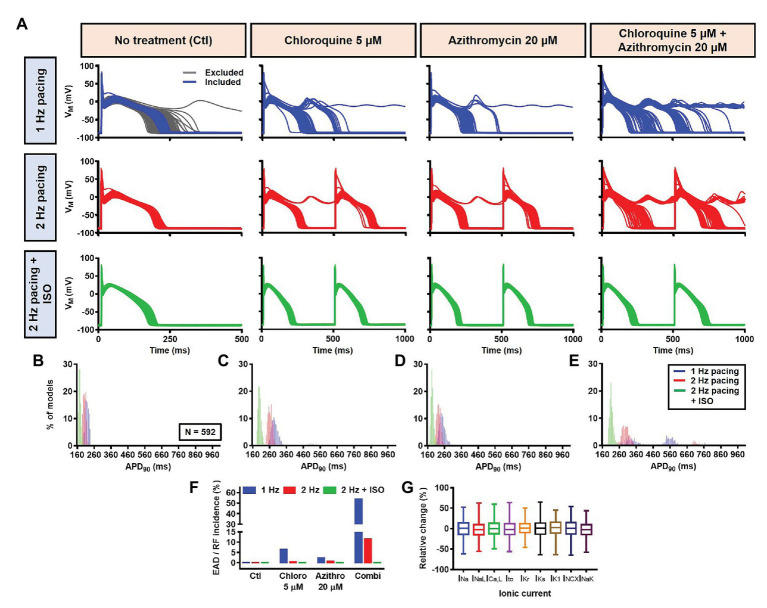
The cellular effects of CQ and AZM in the population of 1,000 canine ventricular epicardial myocyte models. **(A)** The APs of 592 models included in the study, with the 408 excluded non-physiological APs shown as gray lines. **(B–E)** The frequency distribution of APD in non-treated, CQ, AZM and combined groups. **(F)** The incidence of EAD/repolarization failure (RF) observed in the population-based study (as percentage of models). **(G)** Boxplot showing the distribution of relative changes in ionic currents to accommodate the interindividual variability. AP, action potential; APD, action potential duration; Ctl, control; EAD, early afterdepolarization; ISO, isoproterenol; RF, repolarization failure.

Next, a population-based study was conducted to accommodate intra- and inter-individual variability. A population of 1,000 models was created by varying the conductances of nine ionic currents, as described in the Materials and Methods section. After the exclusion of models with non-physiological baseline APs, 592 models were included in the population (Figure 10A, upper panels, blue lines and Figure 10G). To simulate beta-adrenergic activation, various concentrations of ISO that produced maximum beta-adrenergic response were assigned to each model. Consistent with the default model without variability, at 1 Hz pacing, CQ (5 μM) prolonged the APD by a median 73.3 ms (IQR 67.5–82.3). Similarly, AZM 20 μM prolonged the APD by a median 28.7 ms (IQR 25.5–36.3), and the combination of CQ and AZM prolonged the APD with median 146.5 ms (IQR 92.1–334.7). During 2 Hz pacing, CQ, AZM, and CQ + AZM prolonged the APD with median 59 ms (IQR 56.7–62), 22.7 ms (IQR 20.8–25.5), and 95.2 ms (IQR 84.2–109.3), respectively. Finally, following the addition of ISO, the APD prolongation was further reduced with median prolongation of 26.5 ms (IQR 25.3–27.9) in CQ, 13.5 ms (IQR 13.1–14.2) in AZM, and 37.6 ms (IQR 35.7–40.7) in combined groups (Figures 10A–E).

Finally, the incidence of EADs and RF in the population of models was calculated (Figure 10F). During 1 Hz pacing, no EAD/RF was documented in the non-treated group, while 6.4% of models in the CQ group, 2.4% of models in the AZM group, and 53.5% of models in the combined group exhibited EADs/RFs. Following the increase in pacing rate to 2 Hz, the incidence of EAD/RF was reduced to 0.5, 0.7, and 11.5%, respectively. No EAD/RF was observed in any of the groups following the application of ISO during 2 Hz pacing.

### The Effects of Chloroquine and Azithromycin on Human Ventricular Electrophysiology

**Figure 11:**
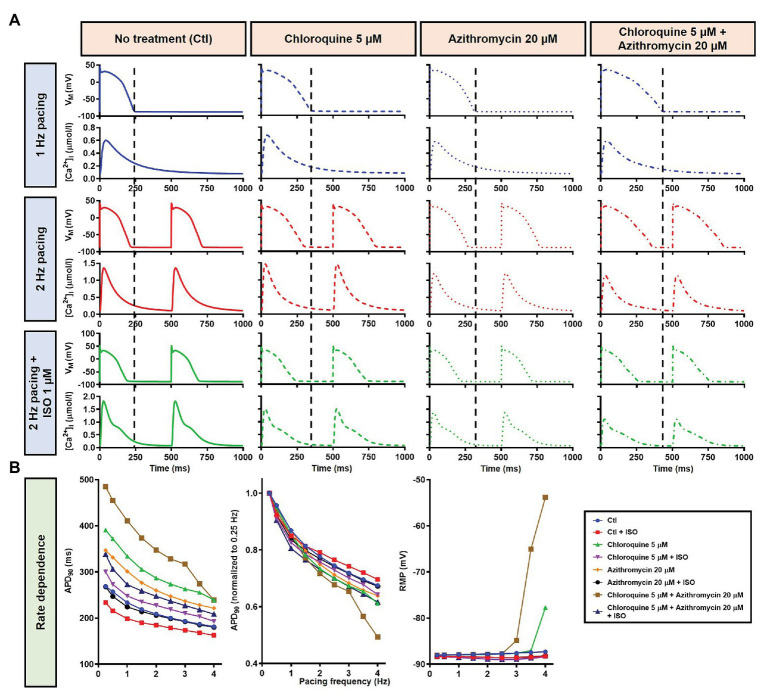
The effects of CQ and AZM on AP properties of human ventricular epicardium. **(A)** The AP and Ca^2+^ transient of non-treated, CQ 5 μM, AZM 20 μM, and combined groups with 1 Hz pacing (blue), 2 Hz pacing (red), or 2 Hz pacing with electrophysiological effects of maximal beta-adrenergic stimulation (green). The dashed vertical lines indicate the end of AP with 1 Hz pacing to provide a clearer depiction of the effects of increasing pacing rate and ISO on APD. **(B)** APD and RMP for different pacing rates in the four groups with and without simulated beta-adrenergic stimulation. AP, action potential; APD, action potential duration; Ctl, control; ISO, isoproterenol; RMP, resting membrane potential.

**Figure 12:**
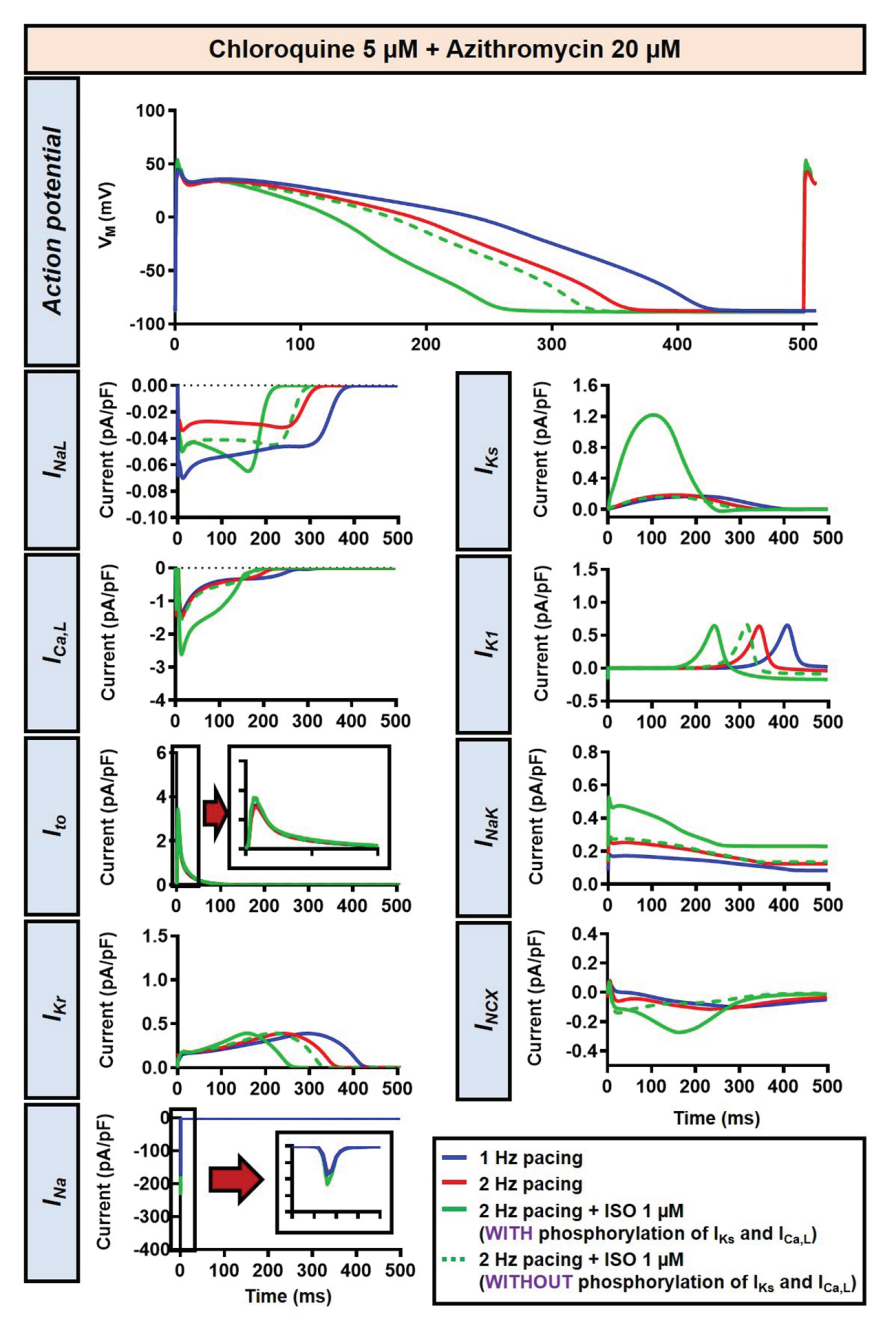
The impact of I_Ks_ and I_Ca,L_ phosphorylation on the action potential of human ventricular epicardium in the presence of CQ and AZM. The effect of CQ 5 μM in combination with AZM 20 μM in the presence of ISO-induced I_Ks_ and I_Ca,L_ phosphorylation is shown in solid green lines. The green dashed lines represent the effect of CQ 5 μM in combination with AZM 20 μM in the absence of ISO-induced I_Ks_ and I_Ca,L_ phosphorylation.

Although the dog is a commonly used animal model with relatively similar electrophysiological properties to humans, some differences in ion-channel expression and AP profile exist, which may modulate drug effects (O’Hara and Rudy, 2012b; Sutanto et al., 2019). As such, we also studied the impact of CQ and AZM on the human ventricular AP. During 1 Hz pacing, application of 5 μM CQ in the ORd human ventricular epicardium model prolonged APD by 100 ms (+43%), while 20 μM AZM prolonged APD by 68 ms (+29%). The combination of both drugs showed a synergistic effect with an APD prolongation of 177 ms (+76%; Figure 11A, upper panels), similar to the canine ventricular model. Likewise, increasing the pacing rate from 1 to 2 Hz reduced the APD in all groups (by 11, 14, 14, and 16% in the non-treated, CQ, AZM, and combined groups, respectively; Figure 11A, middle panels). The combination of simulated ISO application and increased pacing rate reduced APD by 49 ms (−21%) in the non-treated, 106 ms (−32%) in the CQ, 95 ms (−32%) in the AZM, and 164 ms (−40%) in the combined groups compared to APD during 1 Hz pacing (Figure 11A, lower panels). At fast pacing rates, the RMP in the CQ and CQ + AZM groups without ISO displayed a marked depolarization due to incomplete repolarization within a single cycle length (Figure 11B, right panel). Overall, no EAD or RF was observed on the ORd human ventricular model. Similar to the canine ventricular model, ISO-induced phosphorylation of I_Ks_ and I_Ca,L_ contributed to the previously observed APD reduction in the model and preventing such phosphorylation resulted in similar APDs in the CQ + AZM group with 1 μM ISO and the CQ + AZM group at 2 Hz pacing (Figure 12).

**Figure 13:**
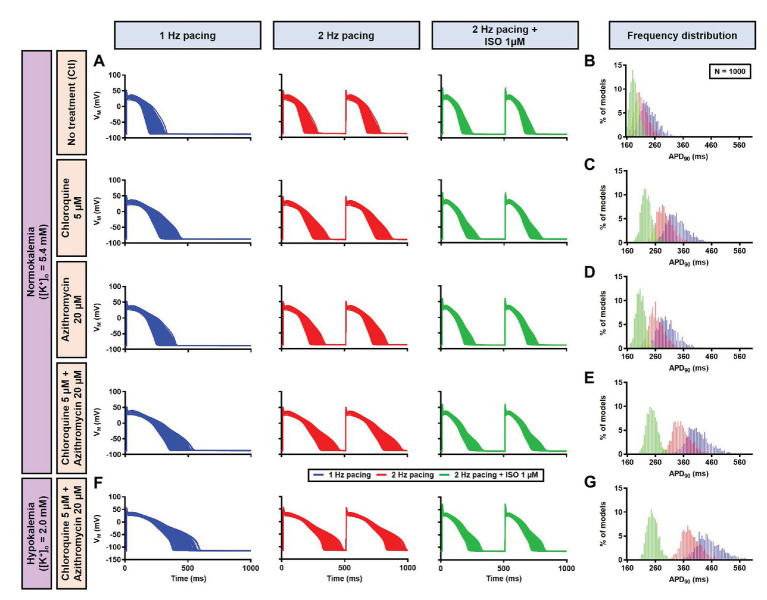
The cellular effects of CQ and AZM in the population of 1,000 human ventricular epicardial myocyte models in the absence and presence of hypokalemia. **(A–E)** The effects of CQ and AZM on the human ventricular myocyte AP in normokalemia ([K^+^]_o_ = 5.4 mM). **(A)** The APs of 1,000 models included in the study under Ctl conditions, with 5 μM CQ, 20 μM AZM, or a combination (top to bottom) at 1 Hz pacing, 2 Hz pacing, or 2 Hz pacing with electrophysiological effects of maximal beta-adrenergic stimulation (left to right). **(B–E)** The frequency distribution of APD in non-treated, CQ, AZM, and combined groups. **(F,G)** The effects of CQ and AZM in the presence of severe hypokalemia ([K^+^]_o_ = 2.0 mM) on human ventricular APs **(F)** and the frequency distributions of APD **(G)**. AP, action potential; APD, action potential duration; Ctl, control; ISO, isoproterenol.

In the population-based study, all 1,000 models were included in the study (Figure 13A, left panels, blue lines). Consistent with the default model without variability, at 1 Hz pacing, APD prolonged by a median 99.4 ms (IQR 94.7–104.8) with CQ (5 μM), 65.4 ms (IQR 61.5–68.9) with AZM (20 μM), and 175.5 ms (IQR 166.2–184.2) with the combination. During 2 Hz pacing, CQ, AZM, and CQ + AZM prolonged the APD with median 79.9 ms (IQR 75.9–83.7), 51.2 ms (IQR 48.8–53.9), and 139.8 ms (IQR 132.9–147.1), respectively. Finally, following the addition of ISO, the APD prolongation was further reduced with median prolongation of 42.4 ms (IQR 39.6–45.3) in CQ, 21.5 ms (IQR 19.7–23.6) in AZM, and 62.3 ms (IQR 57.6–67.6) in combined groups (Figures 13A–E).

**Figure 14:**
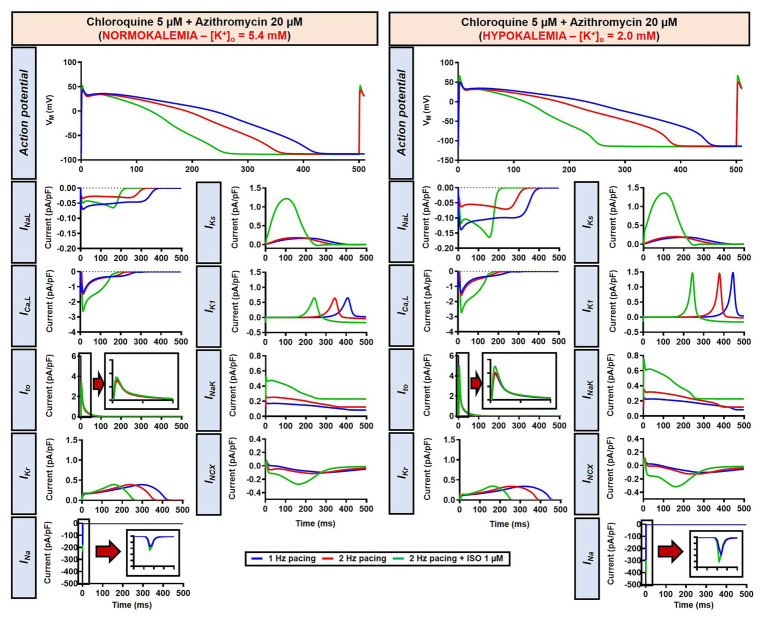
The impact of severe hypokalemia on the action potential of human ventricular epicardium in the presence of CQ and AZM. The **left panels** show the effect of CQ 5 μM in combination with AZM 20 μM in the absence of hypokalemia ([K^+^]_o_ = 5.4 mM). The **right panels** show the effect of CQ 5 μM in combination with AZM 20 μM in the presence of severe hypokalemia ([K^+^]_o_ = 2.0 mM).

To investigate the effect of CQ + AZM on the human AP in the presence of altered repolarization reserve during severe hypokalemia, we lowered the [K^+^]_o_ to 2.0 mM. Severe hypokalemia prolonged the APD while hyperpolarizing the RMP. However, no EAD was induced by this reduction in repolarization reserve (Figure 14). Therefore, we extended the analysis using a population modeling approach to evaluate whether EADs could be induced during hypokalemia in the population of models with variations in ionic current conductance. However, as shown in Figures 13F,G, no EAD was documented in the population of 1,000 models despite the extremely long APD (>600 ms) in some models.

**Figure 15:**
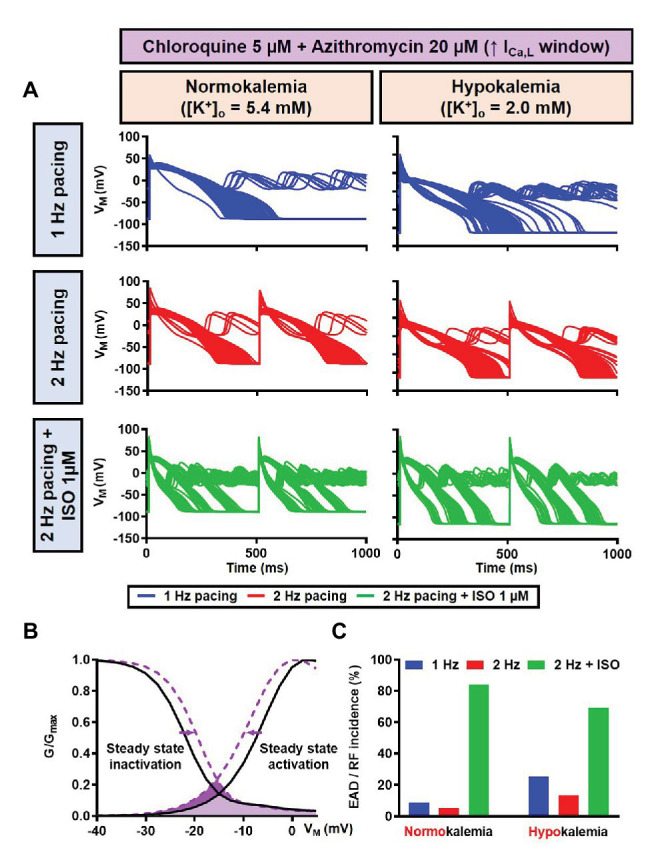
The impact of severe hypokalemia on the population of 1,000 human ventricular epicardium APs with increased I_Ca,L_ window in the presence of CQ and AZM. **(A)** The effect of CQ 5 μM in combination with AZM 20 μM in the absence of hypokalemia ([K^+^]_o_ = 5.4 mM; left panel) and in the presence of severe hypokalemia ([K^+^]_o_ = 2.0 mM; right panel). **(B)** A 3 mV shift in the steady-state activation and inactivation of I_Ca,L_ was introduced in the human ventricular epicardium model to increase the I_Ca,L_ window (shift from black to purple lines). **(C)** The incidence of EAD/RF observed in the population-based study (as percentage of models). EAD, early afterdepolarization; I_Ca,L_, L-type Ca^2+^ current; ISO, isoproterenol; RF, repolarization failure; V_M_, membrane potential.

Since EAD formation has been attributed to the reactivation of I_Ca,L_ (Weiss et al., 2010), we increased the I_Ca,L_ window current by leftward shifting the steady-state activation so that the channels get activated at more negative potentials and rightward shifting the steady-state inactivation so that channels start to recover from inactivation at more positive potentials. Using this approach, we were able to induce EADs in the population of models, both in the absence and presence of severe hypokalemia (Figures 15A,B). Interestingly, in contrast with the EADs resulting from reduced repolarization reserve seen in the canine ventricular model, the EADs generated by the increased I_Ca,L_ window could not be prevented by beta-adrenergic stimulation. As depicted in Figure 15C, while the 2 Hz pacing alone reduced the incidence of EADs and RFs, the application of 1 μM ISO resulted in a marked increase in the EAD/RF incidence, affecting more than 50% of the models. Interestingly, this phenomenon occurred in the presence of ISO-induced APD reduction, highlighting the significance of the I_Ca,L_ window for the generation of EADs.

## Discussion

Here, we investigated the potential proarrhythmic effects of CQ and AZM in the ventricular cardiomyocyte in the absence or presence of beta-adrenergic stimulation using an *in silico* approach. First, both our canine and human models indicate that CQ and AZM can significantly prolong the APD even within their therapeutic range. Moreover, their combination resulted in a synergistic APD prolongation, leading to the initiation of proarrhythmic EADs, which was more pronounced in the presence of reduced repolarization reserve due to reduced extracellular K^+^. Second, beta-adrenergic stimulation reduced APD prolongation in both canine and human models, and prevented EAD formation in the canine model *via* the upregulation of I_Ks_ and I_Ca,L_. Third, our population-based study confirmed the robustness of these findings and showed that beta-adrenergic stimulation completely canceled the initiation of EADs and RFs in canine model variants. Finally, we presented the interesting observation that beta-adrenergic stimulation could increase the incidence of EADs and RFs in the human model with increased I_Ca,L_ window current, highlighting a potential important role for beta-adrenergic activity in modulating drug-induced proarrhythmia by CQ and AZM.

### Chloroquine and Azithromycin Exhibit a Synergistic APD-Prolonging Effect

Chloroquine and azithromycin block multiple ion channels, including the rapid delayed-rectifier K^+^ current (I_Kr_; Crumb et al., 2016), which dose-dependently prolongs the APD and increases the propensity for EADs, creating a substrate for cardiac arrhythmias. In the clinic, they are known to prolong the QT interval, increasing the susceptibility for life-threatening arrhythmias such as torsade de pointes. Anesthetized guinea-pig experiment revealed that CQ indeed has an APD-prolonging effect, while the combination with AZM did not cause further APD prolongation (Fossa et al., 2007). This finding is different from a recent prospective observational study which showed that the maximum corrected QT interval during treatment was significantly longer in the combination group compared to the monotherapy group, highlighting the synergy between CQ and AZM (Saleh et al., 2020), in agreement with our results. Several studies have also reported the potential proarrhythmic effects of CQ and AZM in COVID-19 patients (Jankelson et al., 2020; Saleh et al., 2020; Uzelac et al., 2020; van den Broek et al., 2020). This discrepancy might be due to differences in heart or pacing rate at which the APD-prolonging effects were evaluated as well as interspecies differences in cardiac ion-channel properties, as previously discussed (O’Hara and Rudy, 2012b; Sutanto et al., 2019). Nonetheless, further experiments are needed to confirm the origin of the inconsistency.

Employing a computational canine ventricular cardiomyocyte model, we confirmed the potentially harmful ventricular APD-prolonging effect of CQ and AZM. Within the therapeutic range, the incidence of EADs was relatively low (6.4% in CQ group and 2.4% in AZM group). Nonetheless, the combination of both drugs, as proposed in the treatment of COVID-19, produced a synergistic APD-prolonging effect that further increased the likelihood of EADs, particularly at slow heart rates, suggesting the need for close monitoring of the QT interval during the administration of these drugs in the clinic. The simulations using a human ventricular model also showed a significant APD prolongation, although no EADs were documented. This was in agreement with recent data that reported trivial instances of drug-induced torsade de pointes or arrhythmogenic death following the administration of CQ + AZM despite significant QT prolongation (Chorin et al., 2020; Mercuro et al., 2020; Saleh et al., 2020).

Our results also revealed that hypokalemia further augmented the drug-induced APD prolongation and reduced repolarization reserve. Lowering the [K^+^]_o_ to 4.0 mM produced RF in the canine model without beta-adrenergic stimulation (Figure 9). Similarly, the simulation of severe hypokalemia also prolonged the APD in the human ventricular model, highlighting the importance of baseline repolarization reserve for the proarrhythmic effects of CQ and AZM and the potential for hypokalemia correction to minimize arrhythmia risk in COVID-19 patients under CQ and AZM therapy (Roden et al., 2020).

### Beta-Adrenergic Activation Reduces the APD and Modulates the Cellular Proarrhythmic Risk of Chloroquine and Azithromycin

Beta-adrenergic agonists have been used as an antidote against CQ intoxication for a long time (Don Michael and Aiwazzadeh, 1970; Jaeger et al., 1997). Their benefit in the management of CQ-induced arrhythmia has been experimentally demonstrated in anesthetized rats, showing that the CQ-infused group treated with isoprenaline (a selective beta-adrenergic receptor agonist) displayed longer time to the onset of arrhythmias and death (Buckley et al., 1996). Conversely, the administration of propranolol (a beta-adrenergic receptor blocker) potentiated the electrocardiographic effects of CQ, indicating that beta-adrenergic receptor blockade might render the heart more vulnerable to the actions of CQ (Sofola, 1983).

In this study, we demonstrated that beta-adrenergic stimulation could be a potential protective factor against CQ- and AZM-induced proarrhythmia by lowering the APD prolongation and, therefore, preventing the occurrence of afterdepolarizations. Our population-based study using cellular models of canine ventricular electrophysiology showed that the protective effects of beta-adrenergic stimulation are robust, reducing the incidence of EADs and RFs for a large number of virtual genotypes and with a relatively wide range of simulated isoproterenol concentrations. Similar APD-reducing effects of beta-adrenergic stimulation were also demonstrated in the population of human ventricular models, restoring the drug-induced reduction in repolarization reserve. We also showed that the APD-reducing effect of beta-adrenergic stimulation was due to the PKA-mediated phosphorylation of I_Ca,L_ and I_Ks_. Phosphorylation of I_Ks_ increases the current density during beta-adrenergic stimulation, promoting repolarization. Meanwhile, phosphorylation of I_Ca,L_ abbreviated APD through the elevation of the plateau potential due to increased I_Ca,L_, promoting additional voltage-dependent activation of repolarizing K^+^ currents (e.g., I_Ks_; Johnson et al., 2013), as demonstrated in Figure 6. Moreover, stronger Ca^2+^-dependent I_Ca,L_ inactivation (CDI) during beta-adrenergic stimulation due to increased Ca^2+^ loading (Figures 3A, 11A, lower panels), together with I_Ca,L_ phosphorylation, may give larger peak I_Ca,L_ current density but also a reduction in the persistent, APD-prolonging component. Genetic mutations altering I_Ks_ phosphorylation, as reported in long-QT syndrome type 1, may be responsible for prolonging the AP with beta-adrenergic stimulation, especially at slower cycle lengths (Heijman et al., 2012; O’Hara and Rudy, 2012a; Bartos et al., 2014). Although extrapolation of these findings to the clinical setting is challenging, they suggest that the concomitant sympathetic stimulation in COVID-19 patients may reduce the likelihood of drug-induced torsade de pointes or arrhythmogenic death in COVID-19 patients despite the presence of marked QT interval prolongation, in line with observational studies (Saleh et al., 2020).

On the other hand, our results suggest that beta-adrenergic stimulation might also be harmful in the presence of increased I_Ca,L_ window current. Such conditions may arise from gain-of-function mutations in the L-type Ca^2+^ channel (LTCC)-encoding genes, underlying e.g., long-QT syndrome type 8 (Timothy syndrome; Boczek et al., 2015). Increased I_Ca,L_ window current has also been reported in failing human and rat ventricular myocytes, where a redistribution of functional LTCCs, increased open probability, and CaMKII-mediated phosphorylation of the channel occurred (Sanchez-Alonso et al., 2016). In our *in silico* analyses, isoproterenol-induced EAD promotion occurred irrespective of the APD-reducing effect of beta-adrenergic stimulation, indicating the importance of I_Ca,L_ reactivation in maintaining EAD. This finding suggests the need for careful consideration of beta-adrenergic stimulation under disease conditions that potentially enlarge the I_Ca,L_ window. Moreover, long-term beta-adrenergic stimulation promotes cardiac remodeling, including hypertrophy, fibrosis, and the downregulation of several ion channels *via* transcriptional and post-translational modifications, potentially creating a substrate for cardiac arrhythmias (Scheuer, 1999; Dang et al., 2020; Sutanto et al., 2020b). The present study revealed that transient activation of the beta-adrenergic response may be beneficial against drug-induced proarrhythmia and beta-blockers might not be appropriate under such circumstances. On the other hand, beta-blockers could be used to reduce the detrimental effect of long-term beta-adrenergic stimulation or to reduce the complications of COVID-19-induced systemic inflammation in the absence of medications with proarrhythmic behavior.

### Limitations of the Study

Here, we performed a computational study using an established canine ventricular cardiomyocyte model with beta-adrenergic signaling (Heijman et al., 2011; Tomek et al., 2017) and a human ventricular cardiomyocyte model (O’Hara et al., 2011), phenomenologically incorporating the maximum electrophysiological effect of beta-adrenergic stimulation (O’Hara and Rudy, 2012a; Gong et al., 2020). Despite similarities between canine and human electrophysiology, future studies integrating all signaling components of beta-adrenergic cascades in a human cardiomyocyte model are warranted. Although EADs are an established proarrhythmic mechanism, extrapolation of the current findings to tissue- or organ-level simulations, taking into account the heterogeneous nature of sympathetic innervation, would be required to confirm the pro- and antiarrhythmic effects identified at the cellular level and to identify potential markers of proarrhythmic risk. These were not performed due to the computational costs associated with the complexity of the cardiomyocyte model and the relatively slow time-course of beta-adrenergic stimulation-induced electrophysiological modulation (requiring long simulations).

In this study, we used cellular concentrations of CQ and AZM, i.e., concentrations employed during *in vitro* experiments where specific drugs produce certain measured effects at the cellular level. However, it can be challenging to correlate these cellular concentrations to the clinically relevant doses due to variability in the pharmacokinetics and dynamics of the drugs, particularly in severely ill patients. Pharmacokinetics/dynamics models exist and, in the future, could be implemented to obtain a more precise simulation of the electrophysiological consequences of the drugs.

The drug-induced ion-channel modifications incorporated in this study (Figure 2) were derived from a previous publication using heterologous expression systems (in Chinese hamster ovary/human embryonic kidney cells), which could display different results from human cardiomyocytes (Crumb et al., 2016). Since these data are the only available data to date, we assumed that the relative drug effects are retained across species and cell types.

## Conclusion

Chloroquine and azithromycin exhibit synergistic APD-prolonging effects, potentially resulting in increased proarrhythmic risk, although the severity of the electrophysiological effects depends on the baseline repolarization reserve. Transient activation of the sympathetic nervous system may prevent CQ- and AZM-induced proarrhythmia by reducing their APD-prolonging effect, highlighting the importance of preserving beta-adrenergic response in the presence of such proarrhythmic medications and the potential significance of heart-rate and autonomic-status monitoring in particular conditions such as COVID-19.

## Data Availability Statement

The datasets presented in this study can be found in online repositories at: www.github.com/jordiheijman.

## Author Contributions

HS and JH conceived the study. HS performed the computational simulations. HS and JH performed the data analysis and drafted the manuscript. All authors critically revised the manuscript and approved the final version.

## Conflict of Interest

The authors declare that the research was conducted in the absence of any commercial or financial relationships that could be construed as a potential conflict of interest.

## Abbreviations

ACE2: Angiotensin converting enzyme type 2
AP: Action potential
APD: Action potential duration
AZM: Azithromycin
Ca^2+^: Calcium
CaMKII: Ca^2+^/calmodulin-dependent protein kinase II
CoV: Coronavirus
COVID-19: Coronavirus disease 2019
CQ: Chloroquine
EAD: Early afterdepolarization
IQR: Interquartile range
ISO: Isoproterenol
K^+^: Potassium
PKA: Protein kinase-A
PKC: Protein kinase-C
RF: Repolarization failure
RMP: Resting membrane potential
ROS: Reactive oxygen species
SARS: Severe acute respiratory syndrome
